# Bias of the Immune Response to *Pneumocystis murina* Does Not Alter the Ability of Neonatal Mice to Clear the Infection

**DOI:** 10.3390/jof7100827

**Published:** 2021-10-02

**Authors:** Cathryn Kurkjian, Melissa Hollifield, David J. Feola, Beth A. Garvy

**Affiliations:** 1 Department of Microbiology, Immunology, and Molecular Genetics, College of Medicine, University of Kentucky, Lexington, KY 40536, USA; Kurkjiancj@gmail.com (C.K.); Melissa.Hollifield@uky.edu (M.H.); 2 Department of Pharmacy Practice and Science, College of Pharmacy, University of Kentucky, Lexington, KY 40536, USA; david.feola@uky.edu; 3 Division of Infectious Diseases, Department of Internal Medicine, College of Medicine, University of Kentucky, Lexington, KY 40536, USA; 4 Veteran’s Affairs Medical Center, Lexington, KY 40502, USA

**Keywords:** neonate, lung, infection, mice, *Pneumocystis*

## Abstract

Newborn mice are unable to clear *Pneumocystis* (PC) infection with the same efficiency as adults due, in part, to their inability to develop a robust immune response to infection until three weeks of age. It is known that infants tend develop a Th2 skewed response to antigen so we sought to determine whether a biased cytokine response altered the clearance of PC infection in neonatal mice. *P. murina* infection in neonatal mice resulted in increased IL-4 expression by CD4 T cells and myeloid cells, augmented IL-13 secretion within the airways and increased arginase activity in the airways, indicative of Th2-type responses. *P. murina*-infected IL-4Rα^−/−^ neonates had a shift towards Th1 cytokine production and increased numbers of CD4 and CD8 T cells within the lung as well as elevated levels of *P. murina*-specific IgG. IFNγ^−/−^ and IL-23 p19^−/−^ mice had altered CD4-T cell-dependent cytokine and cell responses. Though we could alter the T helper cell environment in neonatal knockout mice, there was no loss in the ability of these pups to clear infection. It is possible that the Th2 phenotype normally seen in neonatal mice protects the developing lung from pro-inflammatory immune responses without compromising host defense against *P. murina*.

## 1. Introduction

PC is an opportunistic fungal pathogen that is the causative agent of *Pneumocystis* Pneumonia (PCP) in immunocompromised hosts. Primarily known as an AIDS-defining illness, PCP outbreaks were first described among malnourished children within orphan homes [1,2]. An early primary exposure to PC is evident within the population, as a large portion of children 2–3 years of age has been found to make antibody specific to PC antigens [3,4,5,6,7]. Healthy infants are susceptible to PC carriage, likely because of ongoing post-natal lung development and immaturity of the immune system [6,7,8]. We have shown in mouse models that neonates fail to mount an immune response to PC comparable to that seen in adults [9,10] likely due to an inexperienced adaptive immune system as well as an inherent inability to develop a robust innate immune response to the infection [11,12].

Clearance of PC requires a functional CD4 T cell response coupled with activation and response of B cells and macrophages [13,14,15,16,17]. We previously demonstrated in a murine infection model that mice are unable to mount an immune response to PC until 3 weeks of age, resulting in delayed resolution of infection as compared to adults [9,10,18,19,20,21,22,23]. This corresponds to delayed T cell infiltration into airways, delayed macrophage infiltration and activation, and delayed cytokine production [9,10,19,21,22]. T cells isolated from draining lymph nodes of pups, however, are capable of producing IFNγ and proliferating in response to anti-CD3 stimulation [18], thereby suggesting that deficiencies in the neonatal immune response to PC also reside within innate immune defenses. Neonatal alveolar macrophages (AMs), in fact, do display intrinsic defects, as they are unable to translocate NFκB (p65) in response to in vitro stimulation with PC [23]. Augmented levels of TGFβ within the developing neonatal lung [23] may affect the way in which AMs are able to respond to PC within the lung.

T cells from neonates have been shown to respond to various stimuli with a bias toward T helper 2 cytokines in the absence of strong co-stimulation [24,25,26,27]. Th1, Th2, Th17 and Treg responses have all been described during infection to PC [28,29,30,31,32,33,34]. Moreover, in the absence of IFNγ, IL-4, or IL-17 mice are able to clear *P. murina* organisms, albeit with an altered kinetics in some instances [32,35,36,37]. Because the T cell induced cytokine environment drives macrophage activation, it is likely that a heterogeneous macrophage population also exists in the immune response to infection. AMs from healthy humans and mice have been described to have an alternative-like phenotype due to constitutive expression of mannose receptor (MR) and Ym1 [38]. IL-4 and IL-13 mediated activation of AMs drives increased expression of these markers and induction of arginase activity. Because AMs are among the first cells to encounter pathogens within the lungs, an alternative-like phenotype may allow these cells to initially respond to infectious agents without inducing an unnecessary pro-inflammatory response. Data from Nelson et al. demonstrates that alternatively activated macrophages promote clearance of PC in a mouse model of infection [28,39]. We hypothesized that in response to *P. murina* neonatal mice would produce Th2 cytokines leading to alternative activation of AMs that facilitate clearance of the organisms. We indeed found that CD4^+^ T cells produce IL-4 in response to *P. murina* as do a subpopulation of macrophages. IL-4 drove increased arginase activity in the lungs, indicative of alternatively activated macrophages but in the absence of IL-4 and arginase, there was no difference in the ability of infant mice to clear infection. However, IL-4Rα-deficient neonates unable to respond to either IL-4 or IL-13 had increased numbers of activated T cells in the lungs, reduced alternative activation of macrophages, and were able to clear *P. murina* with a slightly faster kinetics than wild type pups. Neonates deficient in IFNγ or IL-23p19 were able to clear *P. murina* with a similar kinetics as wild type mice. These data suggested that a cytokine biased environment significantly effects T cell and macrophage activation and function in response to *P. murina* but has little effect on clearance of organisms.

## 2. Materials and Methods

### 2.1. Mice 

Eight-week-old BALB/c, C57BL/6, B6D2F1, C.129-Il4^tm1Lky^/J (4get), BALB/c-Il4ra^tm1Sz^/J (IL-4Rα^−/−^), B6.129P2-Il4^tm1Cgn^/J (IL-4^−/−^), B6.129P2-Lyzs^tm1(cre)Ifo^/J (LyzM-Cre), and C57BL/6-Arg1^tm1Pmu^/J(Arg^fl/fl^) mice were purchased from Taconic (Germantown, NY, USA) or Jackson Laboratories (Bar Harbor, ME, USA). Interferon γ knockout mice crossed with the Thy1.1/luciferase expressing (originally from Dr. Robert Negrin, Stanford University) (Thy1.1^+^IFNγ^−/−^) and Thy1.1^+^ C57Bl/6 mice were obtained from Dr. Sarah D’Orazio, University of Kentucky [40]. IL-23p19^−/−^ mice (originally from Dr. Daniel Cua, Sherring-Plough/DNAX Corp., San Francisco, CA, USA) were obtained from Dr. John S. Thompson (Lexington, KY, USA, Veterans Administration Medical Center (VAMC)) [41]. Mice were maintained and bred at the Veterinary Medical Unit of the VAMC or the University of Kentucky Department of Laboratory Animal Resources (DLAR) under specific pathogen-free conditions. C.129S6(B6)-Rag2^tm1Fwa^N12 (Rag^−/−^) from Taconic (Georgetown, NY, USA), used to maintain a *P. murina* source colony, were bred and maintained at the VAMC or DLAR in microisolator cages with sterile food and water. Use of animals was approved by the Institutional Animal Care and Use Committees at the University of Kentucky (protocol number: 2010-0692) and Lexington VAMC (protocol number: 2010 09-0010V).

### 2.2. Generation of LyzM-Cre x Arg^fl/fl^ Mice and Genotyping

LyzM-Cre mice were bred to Arg^fl/fl^ mice to generate transgenic mice in which arginase was exclusively deleted from cells specific to the myeloid lineage. Tail snips were performed and DNA was isolated from mice to determine genotype by PCR.

Arginase locus primers: 5′-TgC gAg TTC ATg ACT AAg gTT-3′; 5′-AAA gCT Cag gTg AAT Cgg-3′ (IDT, Coralville, IA, USA). Arginase PCR protocol: 94 °C for 3 min; 35 cycles of: 94 °C for 30 s, 60 °C for 1 min, 72 °C for 1 min; 72 °C for 2 min. PCR products were run on a 3% Tris/Borate/EDTA (TBE) agarose gel and visualized under UV light. The presence of a 250 bp band indicated a mutant locus, 250 bp and 200 bp bands indicated a heterozygote locus, and a 200 bp band indicated a wildtype locus. 

LyzCre locus primers: 5′-CCC AgA AAT gCC AgA TTA Cg-3′; 5′-CTT ggg CTg CCA gAA TTT CTC-3′; 5′-TTA CAg TCg gCC Agg CTg AC-3′ (IDT). LyzCre PCR protocol: 94 °C for 3 min; 35 cycles of 94 °C for 30 s, 62 °C for 1 min, 72 °C for 1 min; 72 °C for 2 min. PCR products were run on a 1.5% TBE agarose gel and visualized under UV light. The presence of a 700 bp band indicated a mutant locus, 700 bp and 350 bp bands indicated a heterozygote locus, and a 350 bp band indicated a wildtype locus.

Mice containing mutant loci for LyzM-Cre and Arg^fl/fl^ were considered to have arginase deleted specifically from myeloid cells and will be referred to herein as Lyz-Arg^−/−^ mice. Arginase activity assays on the lungs of these mice confirmed these results as did PCR on AMs isolated from the bronchial alveolar lavage fluid of the crossed mice. 

### 2.3. P. murina Isolation and Infection 

Lungs were excised from *P. murina*-infected Rag^−/−^ mice and pushed through stainless steel mesh in Hank’s buffered saline solution (HBSS). Cell debris was removed by centrifugation at 100× *g* for 3 min. *P. murina* organisms were enumerated by microscopy as previously described [9]. For inoculation with *P. murina*, mice were anesthetized with isoflurane anesthesia. Eight-week-old adults and 24 to 72 h old neonates were inoculated intranasally (i.n.) with 5 × 10^5^ organisms per gram body weight resuspended in 50 µL or 10 µL of HBSS, respectively.

### 2.4. Isolation of Cells from Alveolar Spaces, Lungs, and Lymph Nodes 

Lung airways were lavaged with 5 exchanges of cold HBSS containing 3 mM EDTA. Bronchial alveolar lavage fluid (BALF) from the first wash was saved for quantitative cytokine analysis. Left lung lobes were snap frozen in liquid nitrogen and stored at −80 °C for quantitative cytokine analysis or arginase assay. Right lung lobes were excised, minced, and digested in RPMI 1640 containing 3% heat-inactivated FCS, 1mg/mL collagenase A (MilliporeSigma, St. Louis, MO, USA), and 50 U/mL DNase (MilliporeSigma) for 1 h at 37 °C in an atmosphere of 5% CO_2_. Digested lungs were homogenized through 70 µm nylon mesh screens to obtain single cell suspensions, and aliquots were taken for enumeration of *P. murina*. Tracheobronchial lymph nodes (TBLN) were excised and pushed through 70 µm nylon mesh screens in HBSS. Erythrocytes were lysed using a hypotonic solution. Cells were washed and counted.

### 2.5. Enumeration of P. murina 

Aliquots of homogenized lungs were diluted, and 100 µL aliquots were spun onto glass slides. Slides were fixed in methanol and stained with Diff-Quik (Siemens Healthcare Diagnostics). *P. murina* nuclei were enumerated by microscopy as previously described [13], and lung burden was expressed as log_10_ nuclei/right lung lobe.

### 2.6. Flow Cytometric Analysis 

For surface staining, lung lavage, lung digest, and TBLN cells were washed with PBS containing 0.1% BSA and 0.02% NaN_3_ (PBA), and 5 × 10^5^ to 1 × 10^6^ cells were subsequently stained with appropriate concentrations of fluorochrome conjugated antibodies against murine T cell markers (CD4, CD8, CD44, CD62L) or macrophage markers (CD11c and CD11b) = Antibodies were purchased from BD Biosciences (Mt. View, CA, USA), Life Technologies, (Waltham, MA, USA), or BioRad (Hercules, CA, USA). 

For detection of intracellular Ym1 (STEMCELL Technologies, Vancouver, BC, Canada), lung lavage and lung digest cells were stained for surface expression of CD11c and CD11b, as described above. Following surface staining, cells were fixed with 10% formalin for 20 min and permeabilized with PBA containing 0.5% saponin. Non-specific binding sites were blocked using an antibody against CD16/CD32 (Life Technologies). Cells were incubated with unconjugated anti-Ym1, followed by incubation with an allophycocyanin (APC)-conjugated anti-rabbit IgG (Jackson ImmunoResearch Laboratories, Inc., West Grove, PA, USA). Rabbit IgG was used as an isotype control (Jackson ImmunoResearch).

For detection of IL-4-eGFP in 4get mice, lung lavage, lung digest, and TBLN cells were stimulated for 2 h with 50 ng/mL PMA and 1 µg/mL ionomycin at 37 °C under an atmosphere of 5% CO_2_, with 10 µg/mL Brefeldin A being added for the last hour of incubation to inhibit cytokine secretion. Cells were stained for surface expression of CD4, CD11c, or CD11b. Expression of cell surface and intracellular markers was determined by multiparameter flow cytometry using a FACSCalibur cytofluorometer (BD Biosciences) or a LSR II (BD Biosciences). Ten to fifty thousand events were routinely acquired for all analyses. Data were analyzed using FlowJo analysis software (BD BioSciences). Gating was done for lymphocytes or large cells using forward and side scatter. 

### 2.7. Cell Sorting 

Lung lavage cells obtained from infected 4get adult and neonatal mice were washed with PBA. For detection of IL-4-eGFP, cells were stimulated for 2 h with 50 ng/mL PMA and 1µg/mL ionomycin at 37 °C under an atmosphere of 5% CO_2_. Brefeldin A (10 µg/mL) was added for the last hour of incubation to inhibit cytokine secretion. Cells were stained for surface expression of CD11b and subsequently sorted based on positive expression of IL-4 and high, mid, or low expression of CD11b. Aliquots of sorted cells were spun onto glass slides, methanol fixed, and stained with DiffQuik. Differential counts were performed by microscopy to enumerate cell types in each population collected. Images were obtained using a Spot digital camera attached to an Eclipse microscope (Nikon Melville, NY, USA).

### 2.8. Cytokine Analysis 

Analysis of cytokines was performed on BALF. TNFα, IFNγ, IL-4, IL-5, and IL-13 concentrations in the BALF were determined by Cytokine Bead Assay (CBA) per the manufacturer’s specifications (BD Biosciences) or ELISA (Life Technologies). Samples analyzed by ELISA were read at a wavelength of 450 nm using a µQuant spectrophotometer equipped with KC Junior Software (Bio-Tek instruments, Inc., Winooski, VT, USA). The CBA was analyzed using a FACS Calibur cytofluorometer (BD Biosciences). Data were analyzed using BD FCAP Array software (BD Biosciences).

### 2.9. Analysis of Arginase Production 

Lung digest cells (1 × 10^6^ to 2 × 10^6^) were resuspended in 100 µL or 200 µL of T-PER lysis buffer (Thermo Fisher, Waltham, MA, USA) containing 10% protease inhibitor (MilliporeSigma) and 10% sodium orthovanadate phosphatase inhibitor (MilliporeSigma), respectively. Arginase activity in lung digest cells was measured as previously described [42]. Briefly, enzyme was activated by addition of 10 mM MnCl_2_ at 55 °C for 10 min. Arginine (0.5 M) was added to samples and incubated at 37 °C overnight. Following incubation, the reaction was terminated by the addition of an acid solution containing H_2_SO_4_, H_3_PO_4_ and ddH_2_0. Samples were then treated with 9% α-Isonitrosopropiophenone diluted in 100% EtOH for 45 min at 100 °C. Samples were allowed to sit in the dark for 10 min prior to read out using a µQuant spectrophotometer (490 nm). Data was analyzed using KC Junior analysis software.

### 2.10. Statistical Analysis 

Data were analyzed using Sigma Plot statistical software (Systat Software, Inc., San Jose, CA, USA). Two way analysis of variance (ANOVA) was used to determine differences between and within groups when appropriate. If assumptions of normality and/or equal variance were not met, each individual time point was analyzed using one way ANOVA, student’s t-test, or nonparametric equivalents Kruskall-Wallis or Mann–Whitney U rank sum tests. If statistical differences were detected by ANOVA or the Kruskall-Wallis test, post hoc tests were run to determine the differences between groups. Data were statistically different when the *p* value was less than 0.05.

## 3. Results

### 3.1. CD4 Lymphocytes Express IL-4 in Response P. murina 

**Figure 1:**
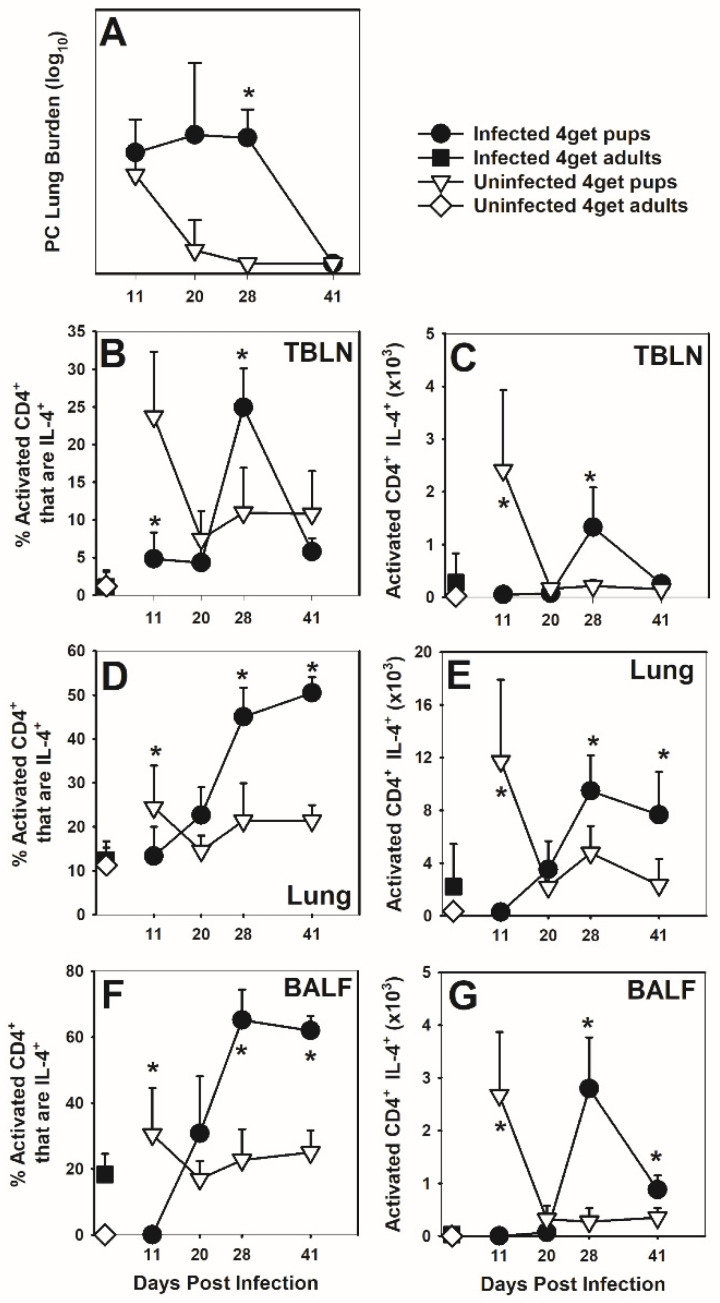
Activated CD4 lymphocytes from *P. murina* infected neonates and adults express IL-4. 4get pups (2-day-old) and adults (≥8 weeks) were infected with 5 × 10^5^ *P. murina* organisms/g of body weight. At indicated days post infection, BALF, right lung lobes, and TBLN were collected from 4–6 mice per group. (**A**) *P. murina* lung burden was determined microscopically. Cells from TBLN (**B**,**C**), lung digest (**D**,**E**), and BALF (**F**,**G**) were treated with PMA and ionomycin, stained with antibody against CD4, CD44 and CD62L, and analyzed by flow cytometry for IL-4-eGFP expression. CD4+ cells expressing high CD44 and low CD62L were considered to be activated. Data represent the mean ± SD and are representative of 2 independent experiments. * *p* ≤ 0.05, comparing *P. murina* infected 4get neonates to *P. murina* infected 4get adults.

**Figure A1:**
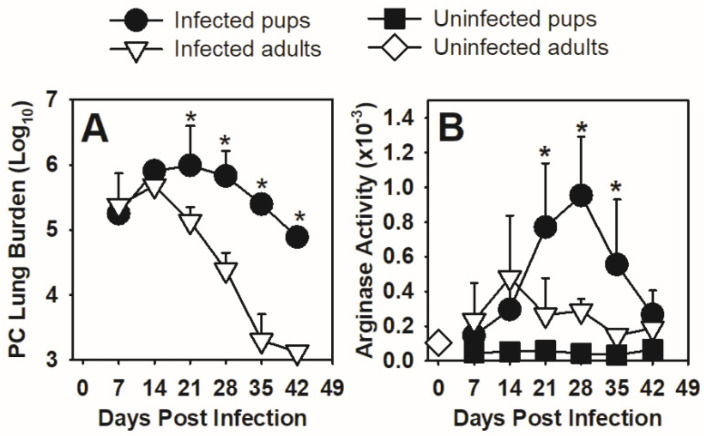
*P. murina*-infected neonatal mice have significantly higher arginase activity in the lungs than infected adults. B6D2F1 mice were infected at 2 days (neonate) or 8 weeks (adults) of age with 5 × 10^5^ *P. murina* organisms/g of body weight. At indicated days post infection lung lobes were collected from 5–6 mice per group. (**A**) *P. murina* lung burden was determined by microscopic quantification of organisms. (**B**) Lung digest lysates were used to determine arginase activity within the lung. Data represent the mean ± SEM from 5–6 mice per group and are representative of 2 independent experiments. * *p* ≤ 0.05, comparing *P. murina* infected adults to infected neonates.

**Figure A2:**
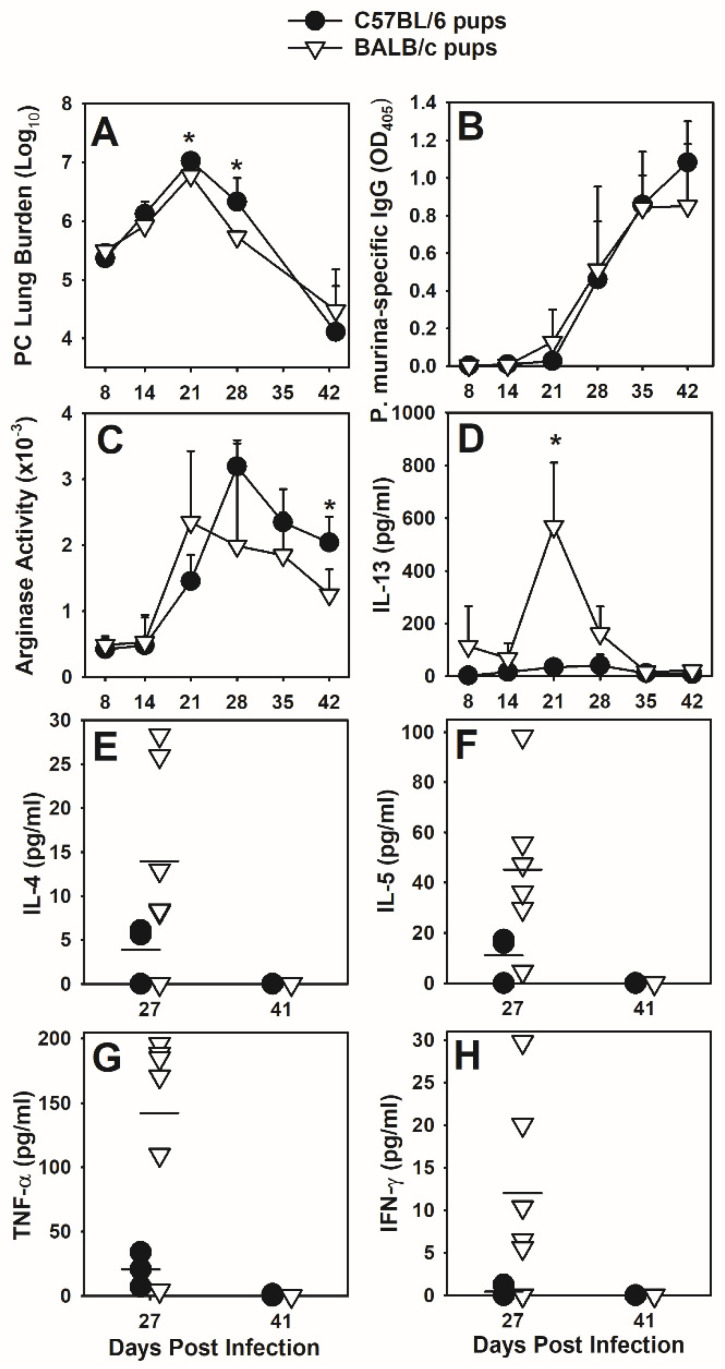
Strain background does not significantly alter clearance kinetics of *P. murina* in neonatal mice. Two-day-old BALB/c and C57BL/6 pups were infected with *P. murina.* At indicated days post infection, serum, BALF, and lung lobes were collected from 5–7 mice per group. (**A**) *P. murina* lung burden was determined microscopically. (**B**) Serum *P. murina*-specific IgG was determined by ELISA. (**C**) Arginase activity was determined from lung digest lysates as described in materials and methods. (**D**) IL-13 within the BALF was determined by ELISA. (**E**–**H**) IL-4, IL-5, TNF, and IFNγ concentration in the BALF were determined by Cytokine Bead Array and are shown as data from individual mice, horizontal line is the mean. * *p* ≤ 0.05, comparing *P. murina* infected BALB/c neonates to *P. murina* infected C57BL/6 neonates.

**Figure A3:**
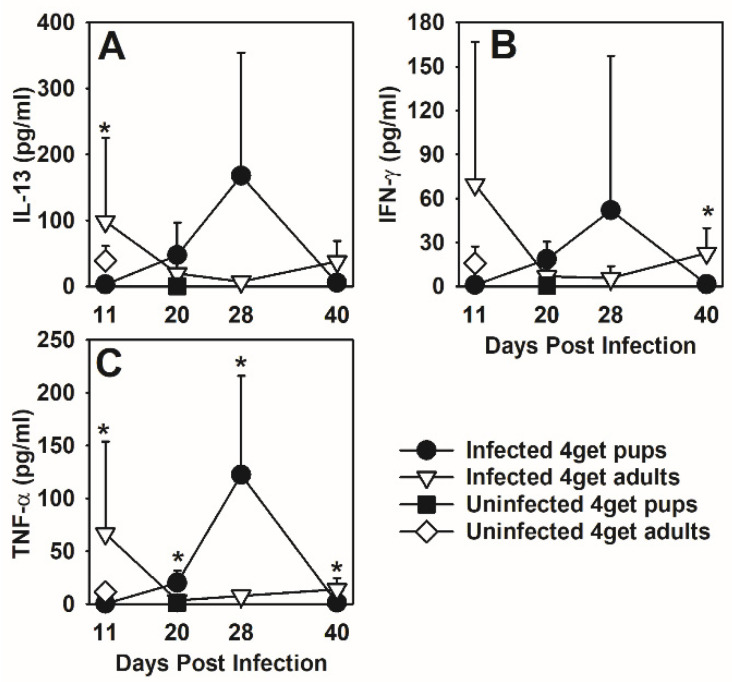
The peak of the cytokine response to *P. murina* is delayed in neonates compared to adults. 4get mice were infected with *P. murina* at 2 days and >8 weeks of age. At the indicated time points, BALF was collected from 4–5 mice per group and assayed for IL-13 (**A**), IFN-γ (**B**), or TNF-α (**C**) concentrations by ELISA. Data represents the mean ± SD and are representative of two separate experiments. * *p* ≤ 0.05 comparing *P. murina*-infected pups and adults at the indicated time point.

Because T cells from neonates are known to have a biased Th2 response to antigens, we first asked whether *P. murina* infection in neonatal mice would result in an IL-4 response in the lungs. We therefore utilized IL-4-eGFP reporter (4get) mice to determine if CD4^+^ T cells produce IL-4 in response to *P. murina*. We have previously reported that adult BALB/c mice cleared *P. murina* between 3 and 4 weeks post-infection while mice infected as neonates did not clear until about 6 weeks post-infection [9]. Transgenic 4get adults and neonates cleared *P. murina* with kinetics similar to that observed in wild type mice on B6D2F1, BALB/c and C57BL/6 backgrounds confirming that the transgene did not impact clearance mechanisms of the organism (Figure 1A, Figure A1A and Figure A2A). The proportion and the absolute number of activated CD4^+^ CD44^hi^CD62L^lo^ cells that also expressed IL-4 peaked in the BALF, lung digest, and draining lymph nodes (TBLN) of adults at day 11 post-infection and in neonates at day 28 or later post-infection (Figure 1B–G). IL-4 expression was not observed in CD8^+^ T cells or B cells within the lungs or TBLN. The peak of IL-13, IFNγ, and TNF levels in the BALF was at day 11 or earlier in adult mice infected with *P. murina* but not until day 28 post-infection in mice infected as pups (Figure A3A–C). These peaks corresponded with the peak of infection for both groups of mice. 

### 3.2. Cells of the Myeloid Lineage Express IL-4 in Response to P. murina 

**Figure 2:**
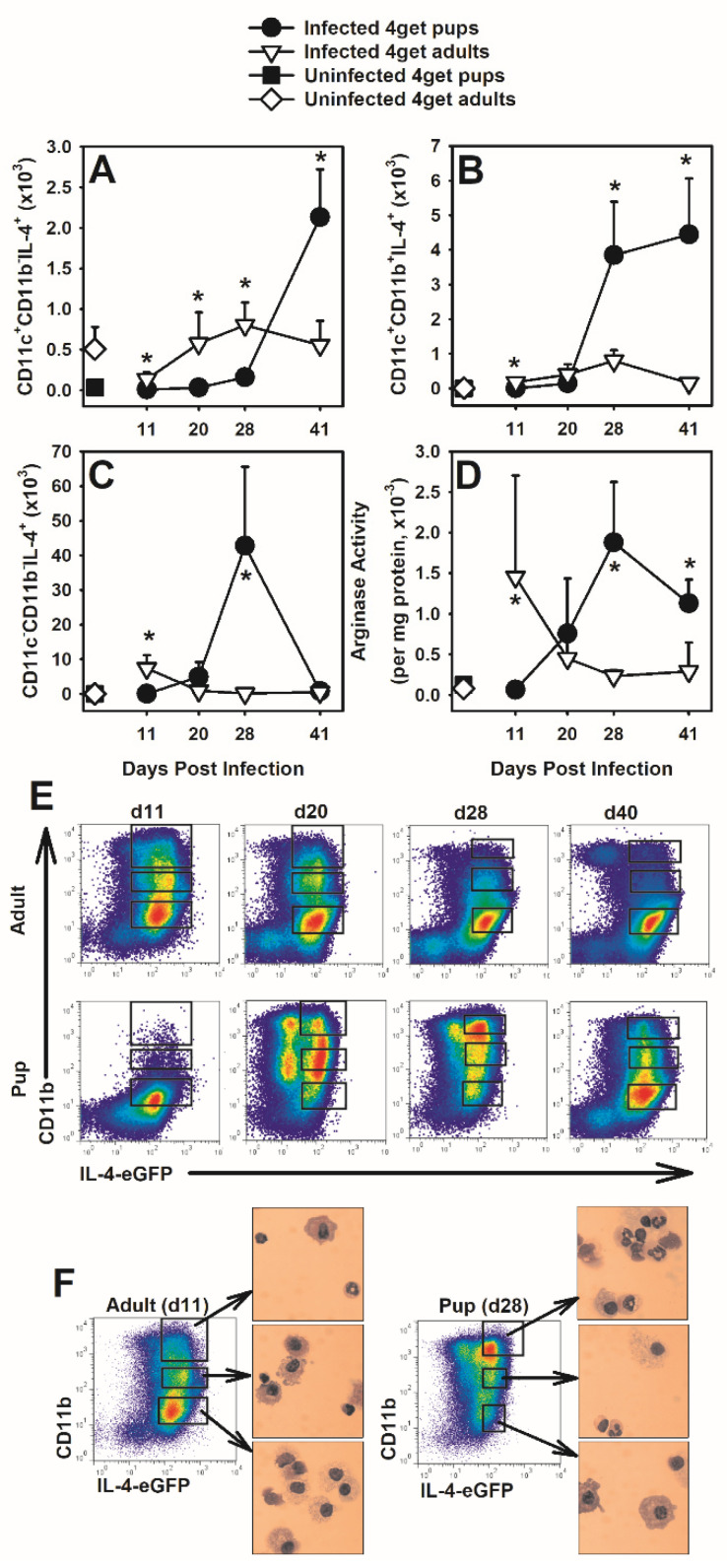
Myeloid cells from BALF of neonates and adults produce IL-4 in response to *P. murina* infection. 4get pups (2-day-old) and adults (≥8 weeks) were infected with 5 × 10^5^ PC organisms/g of body weight. At indicated days post infection, BALF was collected and processed from 4–6 mice per group as described in materials and methods for flow cytometry analysis (**A**–**C**) or sorting of IL-4 expressing cells (**E**,**F**). (**A**–**C**) BAL cells were stained with antibody against CD11c and CD11b and analyzed by flow cytometry. CD11c^+^CD11b^-^ cells are considered to be resident AMs and CD11c^+^CD11b^+^ are considered to be activated macrophages. (**D**) Lung digest lysates were used to determine arginase activity within the lung. (**E**,**F**) BALF cells were stained with antibody against CD11b and sorted based on IL-4 expression and high, intermediate, or low expression of CD11b. Sorted cells were spun onto glass slides and analyzed microscopically based on morphologic characteristics. Data represent the means±SD and are representative of 2 independent experiments. Mann–Whitney rank sum tests were used to compare data at each individual time point. * *p* ≤ 0.05, comparing *P. murina* infected 4get neonates to *P. murina* infected 4get adults.

It has been reported that macrophages can produce IL-4 in response to infection [43]. Following infection with *P. murina*, we observed up-regulation of IL-4 production by the myeloid populations within the BALF of neonatal but not adult mice (Figure 2A–C). Compared to adults, the number of CD11c^+^CD11b^-^ AMs that were IL-4^+^ within the BALF of *P. murina*-infected neonates was significantly increased at day 41 post-infection (Figure 2A). The number of CD11c^+^CD11b^+^ cells (activated AMs) that were IL-4^+^ within the BALF was significantly increased in neonates as compared to adults at day 28 post-infection and remained elevated through clearance of *P. murina* (Figure 2B). Additionally, a significant increase in infiltrating CD11c^-^CD11b^+^ cells expressing IL-4 was observed in neonates at day 28 (Figure 2C). Arginase concentration in the lungs, known to be driven by Th2-like cytokines and produced by alternatively activated macrophages [44], peaked at day 11 post-infection in adult mice and at day 28 post-infection in neonatal mice corresponding to the peak in IL-4-producing cells in the lungs for both groups of mice (Figure 2D). To further characterize these IL-4-producing myeloid cell populations, cells in BALF collected from *P. murina*-infected 4get adults and neonates were sorted based on IL-4 expression and high, intermediate, or low expression of CD11b and visualized by Diff-Quik stain and light microscopy (Figure 2E,F). The morphology of cells within these various populations was similar between adults and pups. Comparing cells at peak infection in adults (day 11) and neonates (day 28), CD11b^mid^IL-4^+^ and CD11b^hi^IL-4^+^ populations had morphological characteristics of both neutrophils and macrophages, whereas the CD11b^lo^IL-4^+^ population was composed of cells with macrophage morphologic characteristics (Figure 2F). Myeloid-derived suppressor cells (MDSCs) have similar morphologic characteristics as both neutrophils and macrophages and have been reported in the lungs of rats and mice with PCP [45]. The total number of myeloid cells producing IL-4 is significantly higher at later time points in *P. murina* infected pups as compared to adults, so it is possible that this cytokine contributes to alternative activation of macrophages and generation of MDSC during the immune response and resolution of infection.

### 3.3. Arginase Is Not Necessary in the Neonatal Immune Response to P. murina 

Elevated arginase activity has been shown to remove L-arginine from the environment resulting in down-regulation of the T cell receptor CD3ζ chain from T cells and is a marker of alternatively activated macrophages [46,47]. Th2 cytokines IL-4 and IL-13, and the immunoregulatory molecule TGFβ, which we have previously shown to be upregulated in postnatal developing lungs infected with *P. murina* [18,23], have been demonstrated to stimulate arginase activity in macrophages [48]. We found arginase activity in the lungs of adults to peak around day 14 post-infection and in neonatal mice between days 21 and 28 following infection with *P. murina* (Figure 2D, Figure A1B and Figure A2C). We did see a small earlier shift in the peak of arginase activity in the lungs of BALB/c pups compared to C57BL/6 pups and significantly reduced levels of IL-13 in the BALF of C57BL/6 pups (Figure A2C,D). The low IL-13 concentrations in C57BL/6 pups did not affect arginase activity which was similar to the BALB/c pups throughout infection. The concentrations of IL-4, IL-5, TNF, and IFNγ in the BALF of BALB/c pups were also higher, though not statistically different, than in C57BL/6 pups at day 27 post-infection (Figure A2E–G). These data may suggest that small local concentrations of IL-4 or IL-13 are sufficient for induction of arginase in AMs in pups.

**Figure 3:**
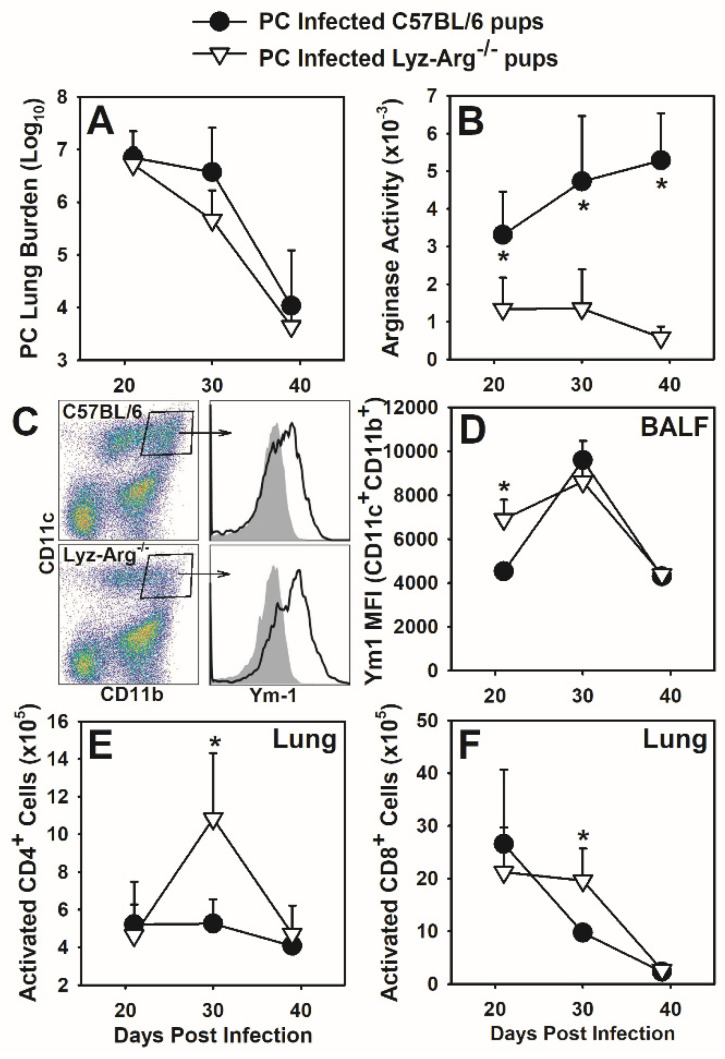
Lyz-Arg^−/−^ neonates have similar immune responses to *P. murina* as wildtype neonates. Two-day-old Lyz-Arg^−/−^ and C57BL/6 (wildtype) pups were infected with *P. murina*. At indicated days post infection, BALF and right lung lobes were collected from 4–6 mice per group. (**A**) *P. murina* lung burden was determined microscopically. (**B**) Lung digest lysates were used to determine arginase activity within the lung. (**C**) BALF cells obtained at day 21 post-infection were stained with antibody against CD11c, CD11b, and Ym1 and analyzed by flow cytometry. CD11c^+^CD11b^+^ are considered to be activated AMs. CD11c^+^CD11b^+^ cells ((**C**), dot plots) were examined for Ym1 expression ((**C**), histograms) and mean fluorescent intensity quantitated as shown in (**D**). (**E**,**F**) Lung digest cells were stained with antibodies against CD4, CD8, CD44 and CD62L. T cell populations expressing high CD44 and low CD62L were considered to be activated. Data represent the mean ± SD and are representative of 2 independent experiments. * *p* ≤ 0.05, comparing *P. murina* infected C57BL/6 neonates to *P. murina* infected Lyz-Arg^−/−^ neonates at the same time point.

To determine if arginase activity has a role in clearance of infection and/or controlling T cell responses in neonates, we utilized neonatal mice on a C57BL/6 background with lysozyme-expressing myeloid cells deficient in arginase (Lyz-Arg^−/−^), and infected them with *P. murina* to observe the immune response and resolution of infection. Lyz-Arg^−/−^ neonates cleared *P. murina* with similar kinetics as wild type C57BL/6 neonates (Figure 3A). Arginase activity was significantly decreased within the lungs of Lyz-Arg^−/−^ neonates as compared to wild type pups suggesting that myeloid cells produce the bulk of the arginase activity in the lungs during infection with *P. murina* (Figure 3B). Examination of macrophage numbers and markers of activation (CD11b expression) and alternative activation (Ym1 expression) did not yield consistent results comparing wild type and Lyz-Arg^−/−^ mice when examined by flow cytometry over two independent experiments (Figure 3C,D). We did find that the absence of arginase in lung myeloid cells did not track with changes in myeloid cell surface markers in infant mice. Following *P. murina* infection, infiltration of activated CD4^+^ and CD8^+^ T cells into the lung parenchyma (Figure 3E,F) and BALF was higher in Lyz-Arg^−/−^ neonates at day 30 post-infection compared to wild type pups. No differences in *P. murina*-specific IgG was seen between wild type and Lyz-Arg^−/−^ mice. Overall, these data demonstrate that myeloid cell-derived arginase does not play a significant role in the resolution of *P. murina* infection in neonatal mice but may help suppress T cell proliferation during the clearance and recovery phase of infection. 

### 3.4. IL-4 Has a Modest Impact on Clearance of P. murina 

**Figure 4:**
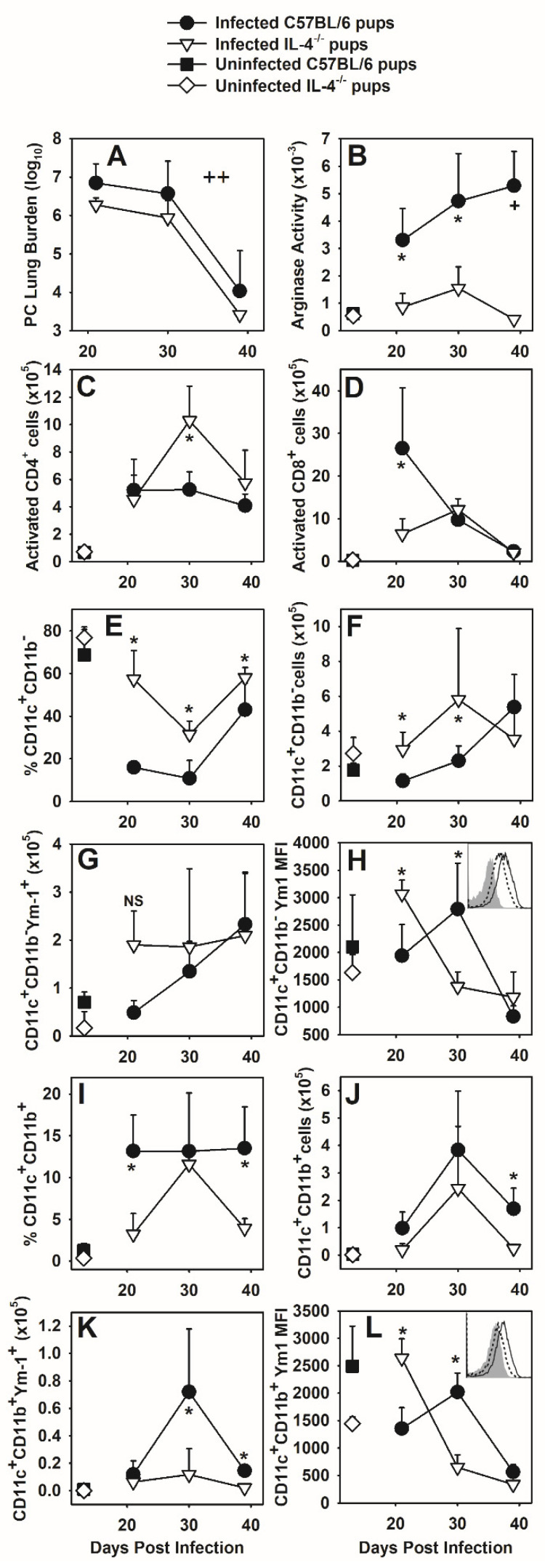
IL-4 dampens the CD4^+^ T cells response to *P. murina* in neonatal mice. Two-day old C57BL/6 and IL-4^−/−^ pups were infected with *P. murina* organisms. At indicated days post infection, BALF and lungs were collected from 4–7 mice per group. (**A**) *P. murina* lung burden was determined microscopically. (**B**) Arginase activity from lung digest lysates was determined as described in materials and methods. (**C**,**D**) Lung digest cells were stained with antibodies against CD4, CD8, CD44 and CD62L. T cell populations expressing high CD44 and low CD62L were considered to be activated. (**E**–**L**) BALF cells were stained with antibody against CD11c, CD11b, and Ym1 and analyzed by flow cytometry. CD11c^+^CD11b^-^ cells are considered to be resident AMs and CD11c^+^CD11b^+^ are considered to be activated macrophages. (**H**,**L**) show representative histograms of Ym1 expression in IL-4^−/−^ (dashed line) or wildtype (solid line) pups at day 30 post-infection. The shaded histogram is an isotype control. The mean fluorescence intensity (MFI) of Ym1 is quantitated over time as shown in the line plots. Data represent the mean ± SD and are representative of 2 independent experiments. * *p* ≤ 0.05; +, *p* = 0.057 comparing *P. murina* infected C57BL/6 neonates to *P. murina* infected IL-4^−/−^ neonates; ++, *p* < 0.05 for the main effect between groups, IL-4^−/−^ pups and wildtype pups irrespective of time points; NS, not significant.

Expression of IL-4 was significantly increased in CD4^+^ T cells (Figure 1) and CD11b^+^ cells (Figure 2) during the neonatal immune response to *P. murina*. Therefore, we infected IL-4 deficient neonates (IL-4^−/−^ on a C57Bl/6 background) to examine the impact of IL-4 on immunity to *P. murina* in infant mice. Overall, IL-4^−/−^ pups had significantly reduced *P. murina* lung burden compared to wild type pups across all time points (main effect), though when we did post hoc statistical tests we found there were not statistically significant differences at any single time point (Figure 4A). The absolute numbers of activated CD4^+^ and CD8^+^ T cells in the lungs of infected wild type and IL-4^−/−^ neonates were similar across most time points, though the peak of infiltration of CD4^+^ T cells around 4 weeks post-infection was consistently up to 2 fold greater in the lung digests of the IL-4^−/−^ pups while the peak in activated CD8^+^ T cells was higher and earlier in wild type pups (Figure 4C,D). These data indicate that IL-4 has a minor inhibitory effect on clearance of *P. murina* as well as on the peak number of activated CD4^+^ T cells in the lungs of infected pups. 

We further examined if the absence of IL-4 affected macrophage activation in the neonatal immune response to *P. murina.* Arginase activity within the lungs of IL-4^−/−^ neonates was significantly decreased as compared to their wild type controls, demonstrating that in response to *P. murina,* IL-4 drives a significant proportion of arginase activity (Figure 4B). The proportion of myeloid cells within the alveolar spaces of infected IL-4^−/−^ neonates was different than in wild type pups, with a higher proportion of CD11c^+^CD11b^−^ cells and fewer CD11c^+^CD11b^+^ cells (Figure 4E,I). The total number of these cell populations followed similar trends (Figure 4F,J) and suggests that IL-4 dampens the transition of resident AMs (CD11c^+^CD11b^−^) to an activation state (CD11c^+^CD11b^+^) [23]. Since IL-4 is associated with driving the transition of macrophages to an alternative activation phenotype, we examined Ym1 expression on CD11c^+^CD11b^−^ AMs and found that the number of Ym1^+^ cells was similar between IL-4^−/−^ and wild type pups however the MFI peaked at day 21 post-infection in IL-4^−/−^ pups compared to a peak at day 30 post-infection in wildtype pups (Figure 4G,H). Similarly, the MFI of Ym1 on CD11c^+^CD11b^+^ cells was significantly decreased as was the number of CD11c^+^CD11b^+^ cells expressing Ym1 at 30 days post-infection in *P. murina* infected IL-4^−/−^ neonates (Figure 4K,L), consistent with Ym1 expression being partially driven by IL-4 in response to *P. murina*. These data indicate that in the neonatal immune response to *P. murina*, IL-4 drives arginase activity and to some extent Ym1 expression. Notably, we did not see any differences in mannose receptor (MR) expression on lung macrophages, another marker of alternative activation. 

**Figure 5:**
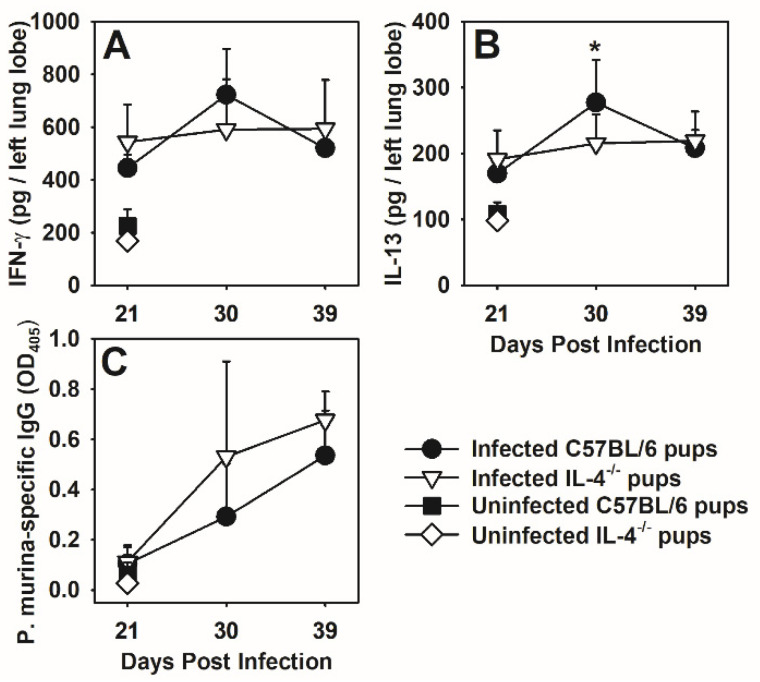
The absence of IL-4 does not significantly alter lung IFNγ levels or *P. murina*-specific serum IgG. Two-day-old C57BL/6 and IL-4^−/−^ pups were infected with 5 × 10^5^ *P. murina* organisms/g body weight. At indicated days post infection, lung homogenates were analyzed for (**A**) IFNγ and (**B**) IL-13 levels in the left lobes by ELISA. (**C**) Serum *P. murina*-specific IgG was analyzed by ELISA. Data represent the mean ± SD of 4–7 mice and are representative of 2 experiments. * *p* < 0.05 compared to IL-4^−/−^ pups at the same time point.

In the absence of IL-4 we found that the levels of IFNγ in pup lungs were not different than in the lungs of *P. murina*-infected C57BL/6 wild type pups after day 21 post-infection (Figure 5A). We also failed to see IL-13 compensate for the lack of IL-4 and in fact IL-13 was lower in the lungs of IL-4^−/−^ pups at day 30 post-infection as compared to wild type pups (Figure 5B). Cytokines in the BALF had similar trends, though the levels were quite low, consistent with the low IL-13 concentrations found in the BALF of C57BL/6 pups as compared to BALB/c pups in supplemental Figure A2D. These data suggest that it is the balance of Th1 and Th2 cytokines, and not the absolute concentrations that control T cell and macrophage responses in pups. 

Because of the changes in T cells, macrophages, and a shift in balance of Th1 and Th2 cytokines in IL-4^−/−^ pups compared to wild type, we considered that *P. murina*-specific IgG could also be altered. However, we found that there was not a difference in the levels of *P. murina*-specific IgG in the sera of C57BL/6 versus IL-4^−/−^ pups at any time point found (Figure 5C). The appearance of *P. murina*-specific IgG was late in the infection in pups, consistent with the delay in organism-specific IgG we have previously reported in the sera of pups compared to adults infected with *P. murina* [9]. 

### 3.5. IL-4Rα Deficiency Alters T Cell Activation in the Neonatal Immune Response to P. murina

**Figure 6:**
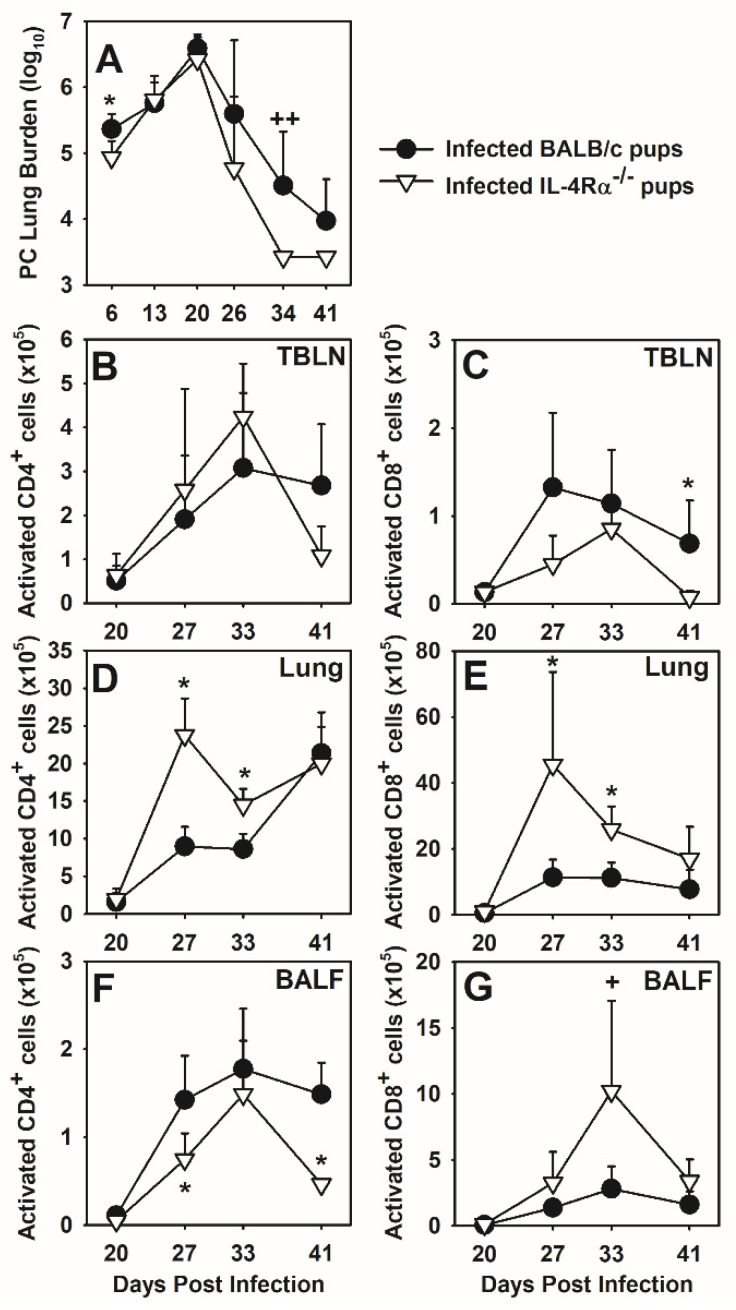
T cells are differentially activated in IL-4Rα^−/−^ neonates infected with *P. murina*. Two-day old BALB/c and IL-4Rα^−/−^ neonates were infected with 5 × 10^5^ *P. murina* organisms/g body weight. At indicated days post infection, TBLN, lungs, and BALF were collected from 4–7 mice per group. (**A**) *P. murina* lung burden was determined microscopically. Cells from TBLN (**B**,**C**), lung digest (**D**,**E**), and BALF (**F**,**G**) were stained with antibody against CD4, CD8, CD44 and CD62L and analyzed by flow cytometry. T cell subsets expressing high CD44 and low CD62L were considered to be activated. Data represent the means ± SD and are representative of 2 independent experiments. * *p* ≤ 0.05; +, *p* = 0.067 comparing *P. murina* infected BALB/c neonates to *P. murina* infected IL-4Rα^−/−^ neonates. ++, organisms burden was below limit of detection for 4 of 4 mice in the IL-4R^−/−^ group and 1 of 4 mice in the BALB/c mice at day 35 and 4 of 4 and 2 of 4 for the IL-4R^−/−^ and BALB/c mice, respectively, at day 41.

IL-4 had a modulatory effect on AMs in neonates infected with *P. murina;* however, we considered that IL-13 could also be contributing to the Th2-like environment in the neonatal lungs. Therefore, we utilized IL-4Rα^−/−^ neonatal mice for our studies since IL-4 and IL-13, though produced, are not able to function through the shared alpha chain of the IL-4 receptor. Notably, these mice were on a BALB/c background with a greater tendency for Th2 responses but little difference in ability to clear infection than C57BL/6 pups as shown in Figure A2. Fifty percent of wild type mice had detectable *P. murina* within their lungs through day 41 post-infection while IL-4Rα^−/−^ neonates completely cleared infection by day 35 post-infection (Figure 6A). There was generally no difference in the absolute number of activated CD4^+^CD44^hi^CD62L^lo^ cells within the tracheobronchiolar lymph nodes (TBLN) of *P. murina* infected IL-4Rα^−/−^ and wild type pups (Figure 6B). The number of CD8^+^CD44^hi^CD62L^lo^ cells in the TBLN tended to be lower in the IL-4Rα^−/−^ pup but the differences were small (Figure 6B). After day 20 post-infection both wild type and IL-4Rα^−/−^ neonates had an influx of activated (CD44^hi^CD62L^lo^) T cells into the lungs, however, IL-4Rα^−/−^ neonates had significantly more activated CD4^+^ cells and CD8^+^ cells within the lungs as compared to wild type neonates at days 27 and 33 post-infection (Figure 6D,E). The number of activated CD4^+^ cells was lower in the alveolar spaces of the IL-4Rα^−/−^ neonates compared to the wild type pups (Figure 6F) while the number of activated CD8 ^+^ T cells was higher in the IL-4Rα^−/−^ pups than in wild type pups (Figure 6G). Overall, it appeared from these data that deficiency in IL-4Rα resulted in a more robust T cell response to *P. murina* infection in the lungs that corresponded to enhanced clearance of the organisms.

### 3.6. Deficiency in IL-4Rα Results in a Reduction in Alternatively Activated Macrophages 

**Figure 7:**
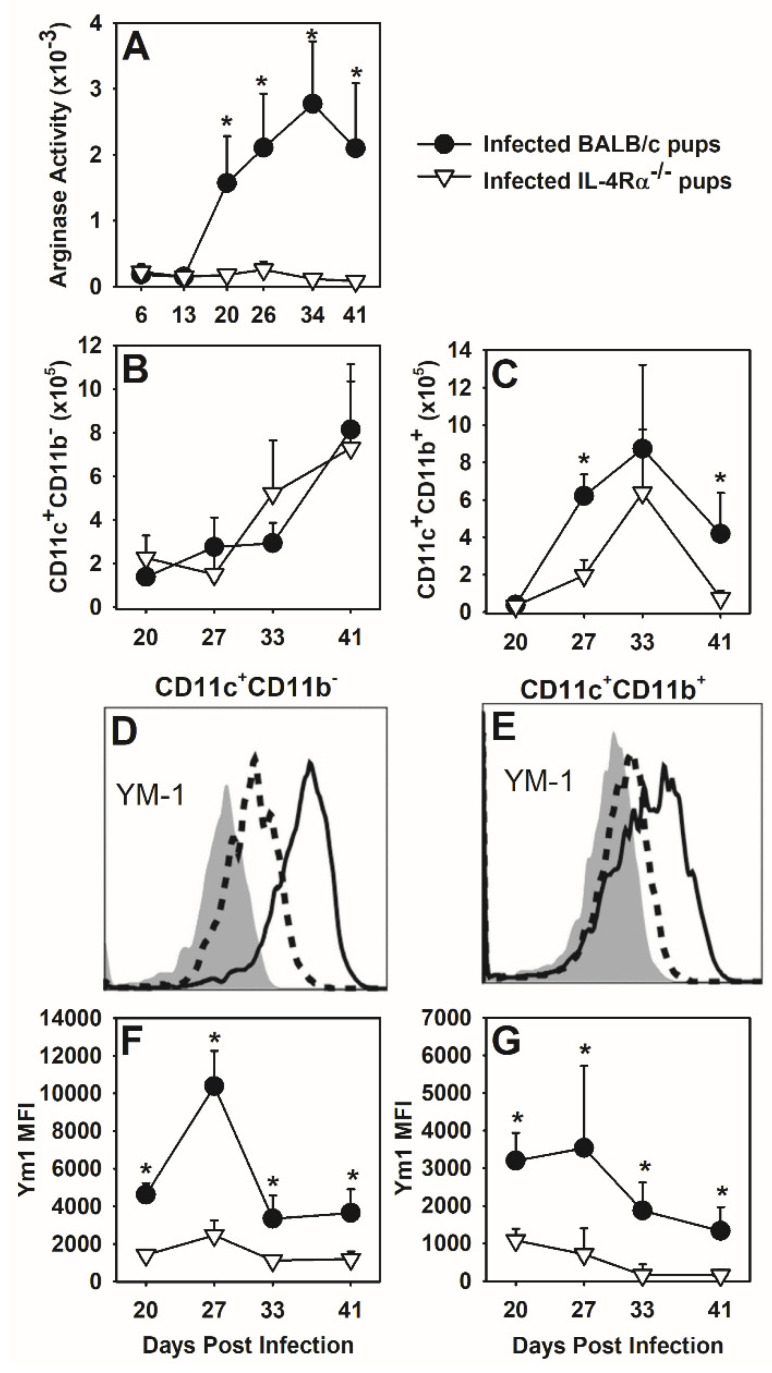
*P. murina* infected IL-4Rα^−/−^ neonates have a reduction in alternatively activated macrophages in the lungs. Two-day old BALB/c and IL-4Rα^−/−^ neonates were infected with 5 × 10^5^ *P. murina* organisms per gram body weight. At indicated days post infection, BALF and lung digest were collected from 4–7 mice per group. (**A**) Arginase activity from lung digest lysates was determined as described in materials and methods. (**B**–**G**) BALF cells were stained with antibody against CD11c, CD11b, and Ym1 and analyzed by flow cytometry. Resident AMs are CD11c^+^CD11b^-^ and activated AMs are CD11c^+^CD11b^+^. Representative histograms from day 27 post-infection depicting Ym1 expression of gated macrophage populations from wild type (solid line) and IL-4Rα^−/−^ (dashed line) mice and an isotype control (shaded) is shown (**D**,**E**). MFI of Ym1 over time is shown in panels F and G. Data represent the mean ± SD and are representative of 2 independent experiments. * *p* ≤ 0.05 comparing *P. murina* infected BALB/c neonates to *P. murina* infected IL-4Rα^−/−^ neonates at the same time point.

We next investigated whether IL-4Rα deficiency in *P. murina* infected neonates affected activation of macrophages. Similar to IL-4^−/−^ neonates (Figure 4B), IL-4Rα^−/−^ neonates had significantly less arginase activity within the lung following *P. murina* infection as compared to wild type neonates, confirming that the ability of macrophages to respond to IL-4 is essential in driving arginase activity during the immune response to infection (Figure 7A). Though there was no difference in the total number of CD11c^+^CD11b^−^ cells (Figure 7B) in the alveolar spaces, IL-4Rα^−/−^ neonates had significantly fewer CD11c^+^CD11b^+^ cells (Figure 7C) in the alveoli at days 27 and 41 post-infection. The expression of Ym1 on CD11c^+^ cells that were either CD11b^−^ or CD11b^+^ was decreased in IL-4Rα^−/−^ pups compared to wildtype as seen by shifts on histograms at day 27 post-infection in Figure 7D,E. The MFI of Ym1 on CD11c^+^CD11b^-^ cells (Figure 7F) and CD11c^+^CD11b^+^ cells (Figure 7G) was significantly lower in IL-4Rα^−/−^ neonates at all days examined post-infection. These data demonstrate that during *P. murina* infection IL-4 and IL-13, signaling through IL-4Rα, drives lung macrophages towards an alternatively activated phenotype as evidenced by expression of Ym1 and production of arginase.

### 3.7. IL-4Rα Deficiency Affects the Cytokine Environment and Antibody Response to P. murina 

**Figure 8:**
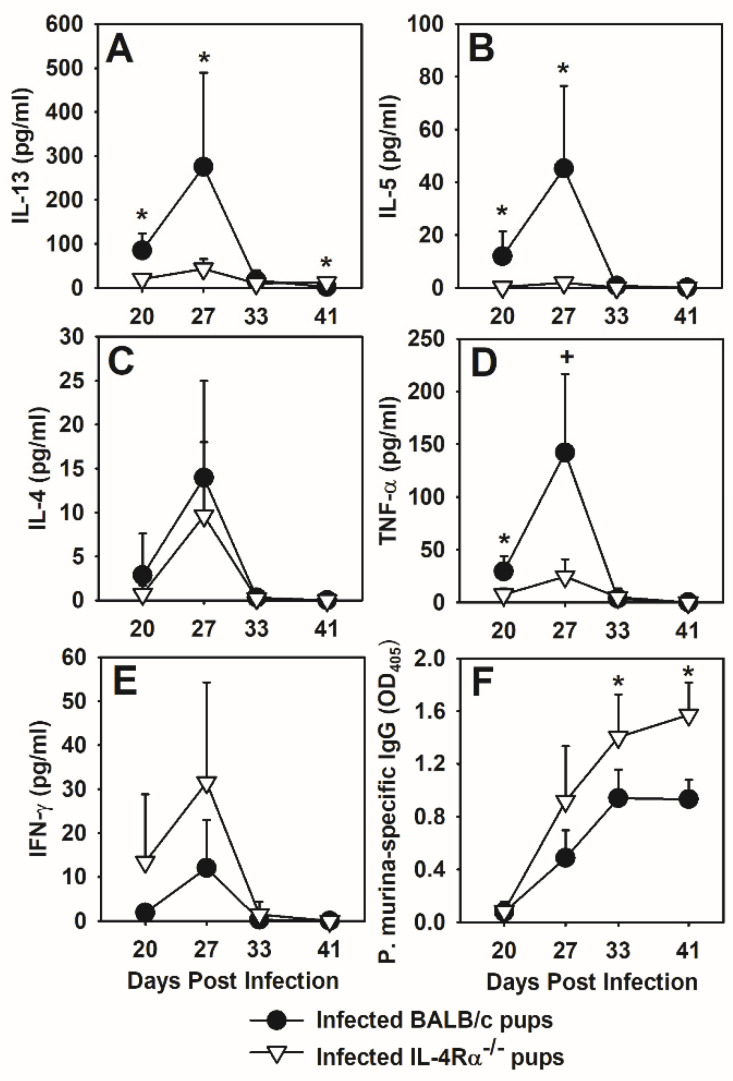
IL-4Rα^−/−^ neonates infected with *P. murina* have a Th1-biased cytokine milieu. Two-day old BALB/c and IL-4Rα^−/−^ neonates were infected with 5 × 10^5^ *P. murina* organisms per gram body weight. At indicated days post infection, BALF was collected from 4–7 mice per group. (**A**) IL-13 in the BALF was determined by ELISA. (**B**–**E**) Levels of IL-5, IL-4, TNFα and IFNγ in the BALF were determined by Cytokine Bead Array. (**F**) Serum *P. murina*-specific IgG was determined by ELISA. Data represent the mean ± SD and are representative of 2 independent experiments. * *p* ≤ 0.05 comparing *P. murina* infected BALB/c neonates to *P. murina* infected IL-4Rα^−/−^ neonates at the same time point.

It is likely that IL-4Rα deficiency alters the cytokine milieu during the immune response to *P. murina* in neonatal mice. We hypothesized that enhanced organism clearance, differential T cell activation, and a change in the phenotype of macrophages in *P. murina* infected IL-4Rα^−/−^ neonates was associated with a decrease in Th2 cytokines and an increase in Th1 cytokines in the lung lavage fluid (BALF). Wild type BALB/c pups had peak levels of IL-13, IL-5 and IL-4 in the BALF at day 27 post infection with *P. murina* (Figure 8A–C). At this time point, *P. murina* infected IL-4Rα^−/−^ neonates had significantly less IL-13 and IL-5 within the BALF as compared to the wild type neonates (Figure 8A,B). There was no difference, however, in IL-4 in the BALF of BALB/c neonates and IL-4Rα^−/−^ neonates at any time point analyzed (Figure 8C). TNFα peaked in the BALF of both strains of mice at day 27 post-infection, however, this cytokine was observed at lower levels in the IL-4Rα^−/−^ neonates (Figure 8D). IFNγ levels in the BALF were inversely related to IL-13 and IL-5, as expected (Figure 8E). While IFNγ increased in the BALF of both strains of mice at day 27 post infection, it tended to be at higher levels in the IL-4Rα^−/−^ neonates (Figure 8E). These data demonstrate that in IL-4Rα^−/−^ neonates, there is a shift in the cytokine milieu during the immune response to *P. murina*.

Although we did not see a difference in *P. murina*-specific IgG in IL-4^−/−^ pups compared to C57BL/6 pups, we considered that the inability of cells to respond to both IL-4 and IL-13 could have an effect on specific antibody production [49]. Indeed, there was a significant increase in *P. murina*-specific IgG in the sera of IL-4Rα^−/−^ pups compared to wild type BALB/c pups (Figure 8F). This increase in specific antibody at days 34 and 41 post-infection corresponded to reduced *P. murina* burden in the lungs of IL-4Rα^−/−^ pups (Figure 6A). Together these data demonstrate that both IL-4 and IL-13 dampens the cellular and humoral immune response to *P. murina* in neonatal mice.

### 3.8. IFNγ or IL-23 p19 Deficiency Alters the Immune Response but Not Ability to Clear P. murina Infection 

**Figure 9:**
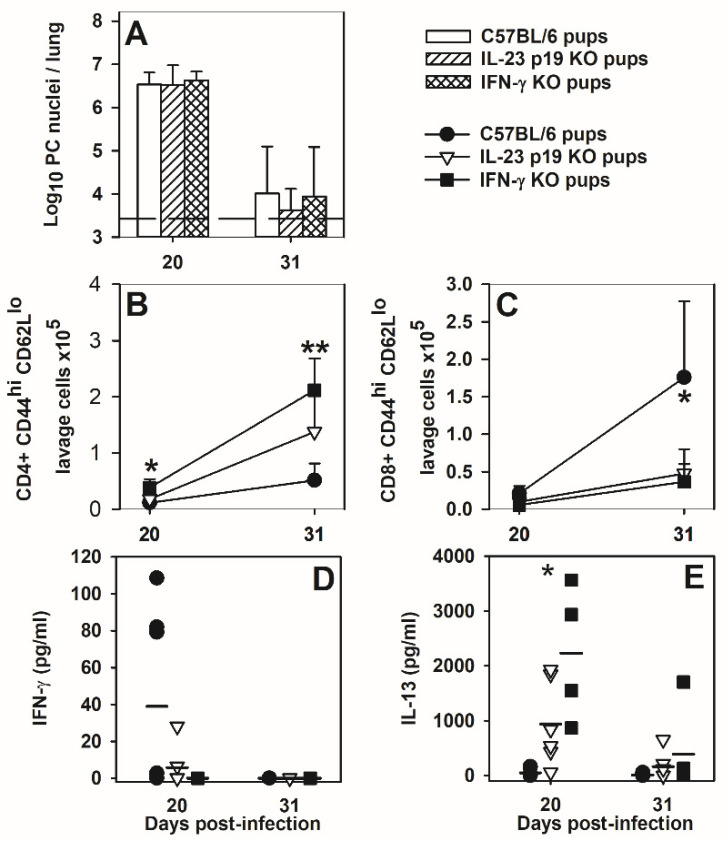
Mice deficient in IFNγ or IL-23p19 clear *P. murina* with the same kinetics as wild type mice. Neonatal mice were infected with intranasal inoculations of 10^6^ organisms and examined at days 20 and 31 post inoculation for (**A**) lung organism burden determined microscopically, (**B**,**C**) = alveolar activated T cell numbers measured by flow cytometry, and (**D**,**E**) cytokine concentrations in the lung lavage fluid measured by ELISA. Data represents the mean ± SD of 4–8 mice (**A**–**C**) or individual mice with the mean shown (**D**,**E**). ** *p* < 0.05 knockout mice compared to C57BL/6 mice, * *p* < 0.05 IFNγ^−/−^ mice compared to C57BL/6 mice.

It is clear from the data shown that though the IL-4 bias effects the characteristics of the neonatal immune response to *P. murina*, IL-4 does not effect the ability of neonatal mice to clear infection. Both IFNγ and Il-17 have been implicated in clearance of *P. murina* in adult mice [32,50], possibly due to their ability to enhance phagocyte function. To determine whether neonatal mice are able to clear *P. murina* infection in the absence of IFNγ or mature IL-17 response, neonates deficient in IFNγ or IL-23 (p19 KO) were examined. As shown in Figure 9A, there was no difference in *P. murina* lung burden between C57BL/6, IFNγ^−/−^, or IL-23 p19KO mice. In the absence of IFNγ or IL-23p19 the numbers of activated CD62L^lo^CD44^hi^ CD4^+^ cells in the alveolar spaces were elevated over the wildtype mice at day 31 post-infection (Figure 9B). In contrast, there were fewer activated CD8^+^ cells in the knockout mice at day 31 post-infection (Figure 9C). IFNγ concentrations in BALF were variable in the wildtype mice and quite low in the IL-23 p19KO mice (Figure 9D). However, IL-13 levels in the lung lavages at day 20 post-infection were significantly elevated in the knockout mice over the wildtype controls consistent with a push toward a more intense Th2 response in the absence of Th1 and Th17 type responses (Figure 9E). Overall, deficiency in IFNγ or IL-23p19 resulted in shifts in lung cells and cytokine levels, but had no effect on clearance of *P. murina*. 

## 4. Discussion

We have previously shown that the ability of neonatal mice to develop an immune response to *P. murina* is significantly delayed as compared to adult mice, resulting in delayed clearance of organisms from the lungs [9,10,18,19,20,21,22,23]. Here, we examined whether Th2 cytokines, known to be abundant in infant mice and humans, have a role in the response to *P. murina* in mice infected as neonates. Because of the known functions of Th2 cytokines, we examined T cell, macrophage, and antibody responses to *P. murina.* We extend our previous findings to demonstrate that neonatal CD4^+^ T cells responding to *P. murina* produce IL-4 later during infection than in adult mice, which corresponds to the delayed infiltration of T cells into the lungs and so delayed clearance of infection. The spike in IL-4 production by T cells and myeloid cells in the lungs occurred at the peak of infection and just prior to the clearance of the infection in pups. In the absence of IL-4 or IL-4Rα pups controlled *P. murina* infection slightly better than wild type pups and had reductions in alternatively activated macrophages at later time points as evidenced by reduced Ym-1 expression and arginase production in the lungs. IL-4^−/−^ and IL4Rα^−/−^ pups had elevated numbers of activated CD4^+^ T cells in the alveolar spaces that corresponded to reduction in organism burden. Cytokine responses were tipped toward Th1 responses not because of elevated IFNγ but because of the absence of IL-4 or reduction in IL-13. There was a more striking reduction in *P. murina* infection in IL4Rα^−/−^ pups at 5 and 6 weeks post-infection than in the IL-4^−/−^ pups which corresponded to significantly increased *P. murina*-specfic serum IgG confirming that both IL-4 and IL-13 dampen the immune response to *P. murina* resulting in a prolonged clearance kinetics.

The cytokine environment elicited by activated CD4^+^ T cells can drive macrophages towards various distinct phenotypes. Cytokines produced by Th1 (IFNγ), Th2 (IL-4, IL-13, IL-33), Th17 (IL-17) and Treg (TGFβ) subsets of CD4^+^ T cells have been observed during the immune response to *P. murina* [28,29,30,31,32,33,34]. Consistent with previous work demonstrating a Th2 bias in neonatal immunity [11,27,51,52,53], we demonstrate a trend towards Th2 biased cytokine production during the neonatal immune response to *P. murina*. IL-4, IL-13, and IL-5, all produced during the immune response to *P. murina* in neonatal mice, are known to drive alternatively activated macrophages [48,54]. Because this phenotype is observed at peak infection and through clearance of organisms from the lung, it is possible that alternatively activated macrophages contribute to resolution of *P. murina.* Consistent with this, the β-glucan receptor Dectin-1 and MR have both been implicated in host defense against PC [55,56] and are both upregulated in IL-4/IL-13 driven alternative activation of macrophages [57,58,59]. Moreover, Myers et al., reported that Th2-biased cytokines and alternatively activated macrophages led to reduced *P. murina* burden in C57BL/6 background STAT4^−/−^ adult mice compared to knockout mice on a BALB/c background [33]. Additionally, serum from HIV-infected individuals colonized with *P. jirovecii* contained significantly lower Th2 cytokine concentrations than HIV-infected patients not colonized with the fungus suggesting that a Th2 cytokine environment is required for protection from colonization with PC [33]. 

Th2 cytokines, IL-13 and IL-33, have been demonstrated to be produced at higher levels than IL-4 during the immune response to *P. murina* in adult mice [28]. Though we have yet to examine a role for IL-33 in the neonatal immune response to infection, we have detected both IL-13 and IL-5 within the BALF of *P. murina* infected BALB/c neonates. IL-13 appears to play a redundant role to IL-4 in driving alternatively activated macrophages in the neonatal immune response to *P. murina.* In the absence of IL-4 or IL-4Rα we found that clearance of organisms is somewhat improved which is counter to this notion that alternatively activated macrophages contribute to defense against *P. murina* and is more consistent with the idea that classically activated macrophages are the key to clearance as evidenced by the long-standing observation that TNF is a critical cytokine for clearance of *P. murina* [60,61,62]. 

We demonstrated that in *P. murina* infected adults and neonates, CD4^+^ T cells and myeloid cells produce IL-4. The production of IL-4 corresponded with sustained alternative activation of AMs during the *P. murina* clearance phase in neonatal mice. Additionally, IL-4-producing myeloid cells also had a phenotype reminiscent of MDSC with both a macrophage and neutrophil-like morphology [45]. MDSC were shown to be present in large numbers in adult rats and mice infected with PC [45]. The MDSC in PC-infected rodents expressed both iNOS and arginase and were able to suppress T cell proliferation [45]. Our observation of the appearance of these cells in neonatal lungs is consistent with the negative regulatory function of IL-4 and may represent a mechanism whereby IL-4 suppresses clearance of *P. murina.*

In the absence of the IL-4Rα we found a significant increase in the production of *P. murina*-specific IgG. This was somewhat surprising since Th2-biased responses are usually thought of as inducing humoral immunity. The elevated specific IgG occurred late during infection and corresponded with the time points at which *P. murina* lung burden was at the limit of detection in the IL-4Rα^−/−^ pups but not in the wild type pups. Previous work has demonstrated that *P. murina*-specific IgG in IL-4^−/−^ mice clears the organisms as or more efficiently than specific IgG in IFNγ^−/−^ mice [36]. Deficiency in IL-4 did not significantly alter *P. murina*-specific IgG, though these mice were on a C57BL/6 background while the IL4Rα^−/−^ mice were on a BALB/c background. While we did not see a difference in the clearance kinetics of *P. murina* on any of the 3 background strains of pups we examined, it may be that IL-13 contributed to the differences we found between IL-4^−/−^ and IL-4Rα^−/−^ pups. In contrast, it has been reported that adult STAT4^−/−^ mice on a C57BL/6 background, with a biased Th2 response, had higher *P. murina*-specific IgG2b and IgG2c titers than wildtype mice while STAT4^−/−^ mice on a BALB/c background had no difference in specific antibody and had higher lung organism burden [33]. The differences in outcome between the Myers et al. study and our data could have to do with the differences in knockout models or alternatively, in the difference in the age of the mice. Indeed, in contrast to neonates, we showed that adult IL-4Rα^−/−^ mice clear *P. murina* with the same kinetics as wild type control mice while LyzM-Arg^−/−^ adults clear *P. murina* with a delayed kinetics compared to wild type control adults [63]. Consistent with this, Zhang et al., found that adult mice lacking IL-4Rα on macrophages were able to clear PC infection with the same kinetics as mice without IFNγR on macrophages concluding that neither M1 nor M2-biased macrophages are critical for host defense against PC [64]. These differences may have to do with intrinsic differences in neonatal macrophages. 

In summary, we have demonstrated that, upon mounting an immune response to *P. murina*, neonatal AMs are driven towards an alternatively activated phenotype, as measured by IL-4 expression in T cells and myeloid cells, IL-13 within the lung environment, and expression of Ym1 and arginase in resident and infiltrating macrophages. Interestingly, MDSC were also found in the neonatal lungs. In IL-4Rα^−/−^ neonates there was a shift from Th2 dominant cytokines towards Th1 cytokines and enhanced clearance of *P. murina*. In contrast, IFNγ^−/−^ or IL-23p19KO pups on a C57BL/6 background, had a shift to a very high IL-13 cytokine response with no effect on clearance kinetics of *P. murina*. While cytokines, such as IFNγ, are important in the resolution of *P. murina*, excess inflammation within developing lungs could cause irreparable damage. Based on these data, we hypothesize that the presence of immunoregulatory cytokines, such as IL-4 and IL-13, and alternatively activated macrophages and MDSC during resolution of infection may play a protective role in the postnatal developing lung. 

## Data Availability

The data presented in this study are shown in the manuscript published here. Raw data are available upon request from the corresponding author.
